# Synaptotagmin 7 switches short-term synaptic plasticity from depression to facilitation by suppressing synaptic transmission

**DOI:** 10.1038/s41598-021-83397-5

**Published:** 2021-02-18

**Authors:** Takaaki Fujii, Akira Sakurai, J. Troy Littleton, Motojiro Yoshihara

**Affiliations:** 1 Memory Neurobiology Project, National Institute of Information and Communications Technology, Kobe, Japan; 2 The Picower Institute for Learning and Memory, Department of Biology, Department of Brain and Cognitive Sciences, Massachusetts Institute of Technology, Cambridge, MA USA

**Keywords:** Short-term potentiation, Synaptic transmission

## Abstract

Short-term synaptic plasticity is a fast and robust modification in neuronal presynaptic output that can enhance release strength to drive facilitation or diminish it to promote depression. The mechanisms that determine whether neurons display short-term facilitation or depression are still unclear. Here we show that the Ca^2+^-binding protein Synaptotagmin 7 (Syt7) determines the sign of short-term synaptic plasticity by controlling the initial probability of synaptic vesicle (SV) fusion. Electrophysiological analysis of *Syt7* null mutants at *Drosophila* embryonic neuromuscular junctions demonstrate loss of the protein converts the normally observed synaptic facilitation response during repetitive stimulation into synaptic depression. In contrast, overexpression of Syt7 dramatically enhanced the magnitude of short-term facilitation. These changes in short-term plasticity were mirrored by corresponding alterations in the initial evoked response, with SV release probability enhanced in *Syt7* mutants and suppressed following Syt7 overexpression. Indeed, *Syt7* mutants were able to display facilitation in lower [Ca^2+^] where release was reduced. These data suggest Syt7 does not act by directly sensing residual Ca^2+^ and argues for the existence of a distinct Ca^2+^ sensor beyond Syt7 that mediates facilitation. Instead, Syt7 normally suppresses synaptic transmission to maintain an output range where facilitation is available to the neuron.

## Introduction

Synaptotagmins (Syts) are a large family of Ca^2+^ binding proteins, with the Syt1 isoform functioning as the major Ca^2+^ sensor for synchronous synaptic vesicle (SV) fusion^1^. Ca^2+^ also controls presynaptic forms of short-term plasticity, with other Syt isoforms representing promising candidates to mediate these processes. Indeed, Synaptotagmin 7 (Syt7) has been reported to function in facilitation^2^, a form of short-term plasticity that enhances synaptic transmission following consecutive action potentials. Facilitation is believed to be mediated by residual Ca^2+^ acting to enhance the number of SVs that are released during repetitive action potentials occurring within a short temporal window^3^. Since Syt7 binds Ca^2+^ with high affinity^4^ and slow kinetics^5^, which match requirements for facilitation, the protein has been hypothesized to act as the Ca^2+^ sensor for this form of presynaptic plasticity. However, the role of Syt7 in facilitation is still unclear.

*Drosophila* neuromuscular junctions (NMJs) provide an excellent system for testing the role of Syt7 in short-term synaptic plasticity. In particular, embryonic NMJs are highly plastic^6^ and allow stable recordings in high Ca^2+^ concentrations using the *myosin heavy chain* (*Mhc*) mutant background to prevent muscle contraction^7^. Together with the lack of compensation that might occur at older synapses, these advantages provide highly reliable measurements of synaptic transmission. Indeed, analysis of *Syt1* mutants at embryonic NMJs established this Syt isoform functions as the synchronous Ca^2+^ sensor for synaptic transmission^8–10^. Although NMJs in many other species show depression, *Drosophila* embryonic NMJs are facilitative at physiological Ca^2+^ concentrations as shown here, similar to many mammalian central synapses such as those in the hippocampus^11^. Using stable recordings from this facilitative and plastic synapse in *Drosophila* embryos, we quantified the absolute value of synaptic currents in *Syt7* mutants and in animals overexpressing Syt7. While loss of Syt7 enhanced presynaptic output, overexpression of Syt7 suppressed release. These changes in the magnitude of presynaptic output were mirrored by changes in short-term presynaptic plasticity. High levels of Syt7 enabled robust facilitative responses while loss of Syt7 switched the normally facilitating synapse into one that displayed short-term depression. This work reveals that Syt7 normally reduces synaptic transmission to scale it to an appropriate range where facilitation is allowed, providing a bi-directional switch for short-term synaptic plasticity.

## Results

### Syt7 switches short-term synaptic plasticity from depression to facilitation

**Figure 1:**
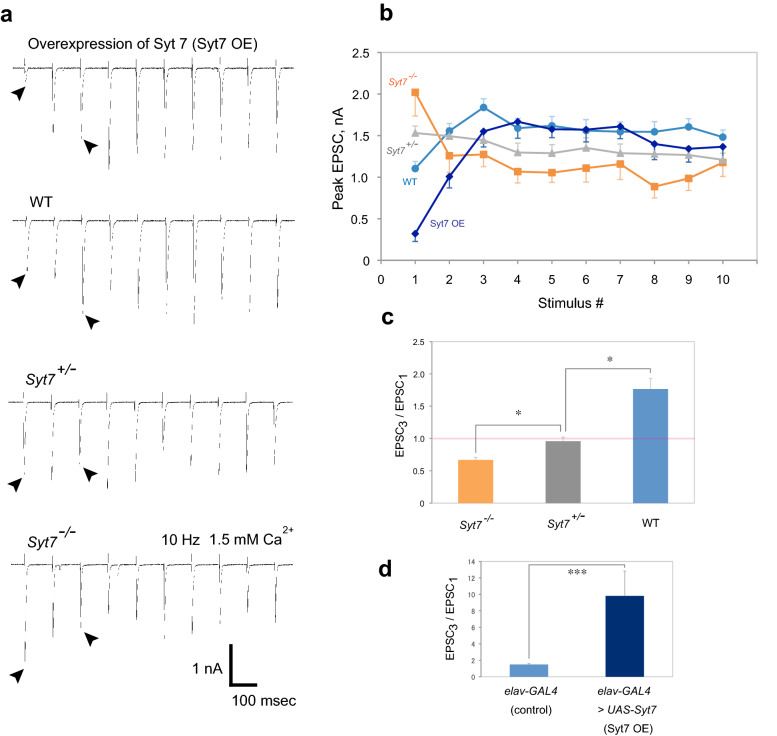
Syt7 switches short-term plasticity from depression to facilitation. (**a**) Representative traces of synaptic currents in response to 10 pulses of 10 Hz repetitive stimulation at 1.5 mM Ca^2+^ in embryos overexpressing Syt7 (Syt7 OE, *elav-GAL4* > *UAS-Syt7*), WT, *Syt7*^+*/−*^ and *Syt7*^*−/−*^ mutants. Arrowheads indicate comparison of synaptic currents (EPSCs) induced by 1st pulse and 3rd pulse. (**b**) Quantification of 10 Hz stimulation experiments. The number of recorded cells analyzed for each genotype: Syt7 OE, 9; WT, 13; *Syt7*^+*/−*^, 13; *Syt7*^*−/−*^, 13. (**c**) Facilitation or depression ratios shown as 3rd pulse-induced EPSC (EPSC_3_)/1st pulse-induced EPSC (EPSC_1_). WT, *Syt7*^+*/−*^ and *Syt7*^*−/−*^ mutants were analyzed with the Kruskal–Wallis test using a one-way ANOVA by ranks and significant difference between the groups was found (P < 0.0001). *P < 0.05 by Dunn’s post-hoc multiple comparison test between groups. The red line indicates when no facilitation or depression is observed (ratio = 1). (**d**) Facilitation ratio shown as 3rd pulse-induced EPSC (EPSC_3_)/1st pulse-induced EPSC (EPSC_1_) for *elav-GAL4* controls and Syt7 OE (*elav-GAL4* > *UAS-Syt7*). The results were analyzed with the Mann–Whitney *U* test and significant difference between the groups was found (***P < 0.001). The numbers of recorded cells analyzed for each genotype: *elav-GAL4* , 13; *elav-GAL4* > *UAS-Syt7*, 9. Error bars are SEM.

Whole-cell voltage clamp recordings were performed at muscle fiber 6 in *Drosophila* embryos at hatching stage (21–24 h after fertilization) to record synaptic currents (EPSCs) elicited by stimulation of the glutamatergic motoneurons innervating the muscle. All recordings were done in the background of a null mutation in *Myosin heavy chain* (*Mhc*^1^) to prevent muscle contraction^7^. We stimulated the nerve for 10 pulses at 10 Hz, commonly used in studies of both mammalian central synapses and *Drosophila* NMJs for mimicking natural communication^11^, at the reported physiological concentration of Ca^2+^ (1.5 mM)^12^. As shown in Fig. 1a,b, wild type (WT) embryos display a cumulating increase in response to three consecutive stimuli at the beginning of the 10 Hz stimulation, while *Syt7* null homozygous mutant (*Syt7*^*−/−*^)^13^ embryos show a decreasing response from the larger 1st EPSC than that of WT. The response then reaches a plateau until presumed depletion of the immediate releasable SV pool (IRP) occurs^14^. Strikingly, heterozygotes (*Syt7*^+*/−*^) containing only a single copy of Syt7 showed an intermediate phenotype with almost no facilitation or depression, consistent with the intermediate size of the 1st EPSC compared to WT or *Syt7*^*−/−*^ (Fig. 1a,b). The ratio of the 3rd EPSC to 1st EPSC in Fig. 1c demonstrates switching from depression to facilitation with increasing amounts of Syt7. These results suggest Syt7 normally suppresses synaptic transmission to an appropriate range to prevent depletion of the IRP and ensure synaptic facilitation. To test this hypothesis directly, we overexpressed Syt7 with the elav-GAL4 pan-neuronal driver. Syt7 overexpression suppressed the amplitude of the 1st EPSC compared to WT (Fig. 1a,b) and increased the facilitation ratio by more than sixfold compared with elav-GAL4 controls (Fig. 1d). These results indicate the levels of Syt7 set the initial magnitude of presynaptic output, with normal levels of Syt7 suppressing synaptic transmission to a range where facilitation can occur.

### *Syt7* null mutants show enhanced nerve-evoked transmission with higher release probability than WT

**Figure 2:**
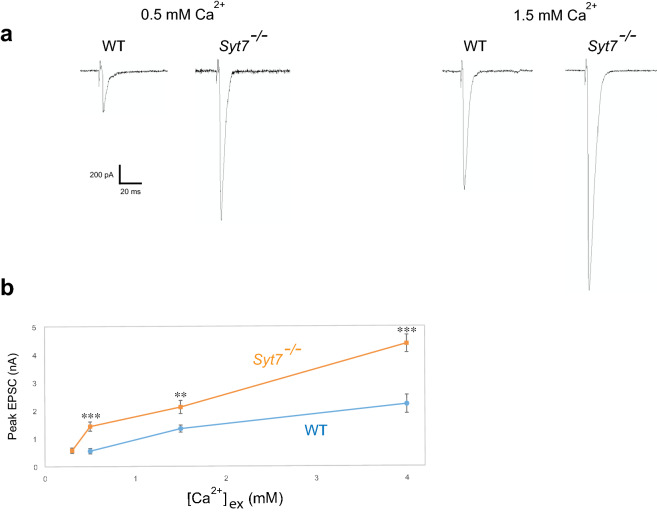
*Syt7* null mutants show enhanced nerve-evoked transmission. (**a**) Representative traces of evoked synaptic currents in wild type (WT) and *Syt7*^*−/−*^ at 0.5 mM Ca^2+^ and 1.5 mM Ca^2+^. (**b**) Ca^2+^ dependence of peak evoked synaptic currents (EPSC). WT and *Syt7*^*−/−*^ were analyzed at each Ca^2+^ concentration with Student’s *t* test, and significant difference between the two groups was found at 0.5 mM Ca^2+^ (***P < 0.001), 1.5 mM Ca^2+^ (**P < 0.01) and 4 mM Ca^2+^ (***P < 0.001). WT was not quantified at 0.3 mM Ca^2+^ due to the high probability of failures. The numbers of recorded cells analyzed for each genotype: (WT) 0.5 mM, 11; 1.5 mM, 11; 4 mM, 8 (*Syt7*^*−/−*^) 0.3 mM, 19; 0.5 mM, 15; 1.5 mM, 20; 4 mM, 9. Error bars are SEM.

**Figure 3:**
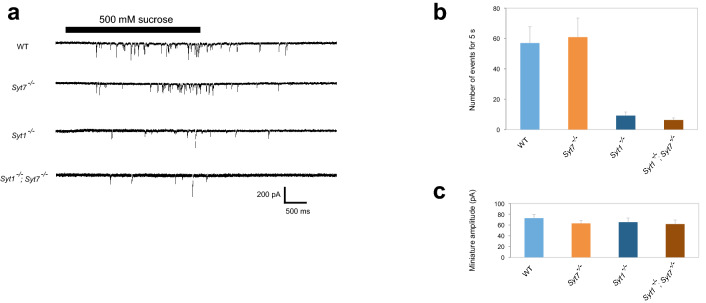
Hypertonic-induced miniature release. (**a**) Representative traces for each genotype. 500 mM sucrose was applied for 3 s (bar) in the absence of Ca^2+^. (**b**) Number of hypertonic-induced miniature synaptic currents recorded during 5 s after the beginning of application of 500 mM sucrose. Numbers of recorded cells: WT, 24; *Syt7*^*−/−*^, 24; *Syt1*^*−/−*^, 38; *Syt1*^*−/−*^; *Syt7*^*−/−*^, 39. (**c**) Amplitude of miniature synaptic currents induced by hypertonic stimulation with 500 mM sucrose. Amplitudes recorded from a single muscle cell were averaged and the averages for each cell are shown. Number of recorded cells are the same as those in b. Error bars are SEM.

We next analyzed individual EPSCs from WT and *Syt7*^*−/−*^. As shown in Fig. 2a,b, at all Ca^2+^ concentrations tested *Syt7*^*−/−*^ mutants show dramatically enhanced synaptic currents that are three-fold larger than WT at 0.5 mM Ca^2+^. Presynaptic changes that can drive larger synaptic responses can be secondary to increased release probability (*P*) of single SV fusion events or an increased number (*N*) of readily-releasable SVs. To differentiate between these two possibilities, we measured the readily-releasable pool size using hypertonic stimulation^15^. As shown in Fig. 3a,b, hypertonic stimulation in *Syt7*^*−/−*^ embryos with 500 mM sucrose solution induced SV release levels similar to WT, indicating mutants do not have a larger releasable SV pool at embryonic synapses. Although the SV pool size is different in *Syt7* 3rd instar mutants^13^, SV pools increase dramatically from embryonic development through the 3rd instar larval stage as synaptic maturation and profound synaptic growth occur. Given the dramatic increase in presynaptic release in embryonic *Syt7* terminals can occur without a change in the releasable SV pool size at this stage of development, release probability for individual SVs is also enhanced following loss of Syt7.

We previously found that *Syt1*^*−/−*^ null mutants have a smaller releasable SV pool than WT^8,9^. We assayed for genetic interactions in SV pool size between *Syt1*^*−/−*^ and *Syt7*^*−/−*^ and found that loss of Syt7 did not change the smaller response in *Syt1*^*−/−*^ mutants (Fig. 3a,b), consistent with enhanced synaptic transmission occurring without an elevated SV pool size in *Syt7*^*−/−*^. We next measured quantal size generated by release of single SVs during hypertonic stimulation, where release is Ca^2+^-independent and does not show multi-quantal release^16^. Quantal sizes of WT and *Syt1*^*−/−*^ are similar to those previously found^8^, and both *Syt7*^*−/−*^ and *Syt1*^*−/−*^; *Syt7*^*−/−*^ double mutants did not alter quantal size (Fig. 3c). These findings indicate there is no defect in the postsynaptic response to SV release in the absence of Syt7.

**Figure 4:**
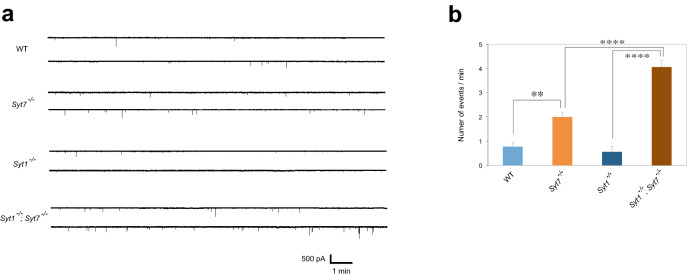
Spontaneous miniature synaptic currents. (**a**) Representative traces for each genotype in the presence of 3 μM Tetrodotoxin (TTX), which inhibits action potentials. Concentration of Ca^2+^ was 1.5 mM. (**b**) Miniature synaptic currents counted during 10 min and shown as number of events per minute for each genotype. The four groups were analyzed with ordinary one-way ANOVA and significant difference between the groups was found (P < 0.0001). **P < 0.01, ****P < 0.0001 by Tukey’s post-hoc multiple comparison test between groups. The numbers of cells: WT, 7; *Syt7*^*−/−*^, 9; *Syt1*^*−/−*^, 5; *Syt1*^*−/−*^; *Syt7*^*−/−*^, 5. Error bars are SEM.

These data indicate the stronger synaptic transmission in *Syt7*^*−/−*^ mutants is likely due to increased *P* rather than changes in *N*. It was previously proposed that Syt1 triggers SV fusion upon action potential-induced Ca^2+^ influx and also clamps fusion at lower [Ca^2+^] to generate a better signal-to-noise ratio^17^. Experimental evidence supporting the clamping model were recorded at *Drosophila* 3rd instar NMJs where miniature frequency in *Syt1*^*−/−*^ is increased compared to WT^18,19^. In the case of embryonic NMJs, we did not detect an elevated frequency of miniature events in *Syt1*^*−/−*^^8,9^. However, *Syt7*^*−/−*^ mutants displayed more frequent miniature release than WT, and *Syt1*^*−/−*^; *Syt7*^*−/−*^ double mutants showed even more frequent miniature release than *Syt7*^*−/−*^ single mutants (Fig. 4a,b). These results support a model where both Syt7 and Syt1 may clamp SV fusion in a synergistic manner as observed at mammalian synapses^20^. We hypothesize that elevated miniature release rate in *Syt1*^*−/−*^ single mutant embryos is cancelled out by the reduced SV pool size at these synapses, as well as its reported role in SV docking and endocytosis^8,9^.

### Syt7 suppresses release probability and enables paired-pulse facilitation

**Figure 5:**
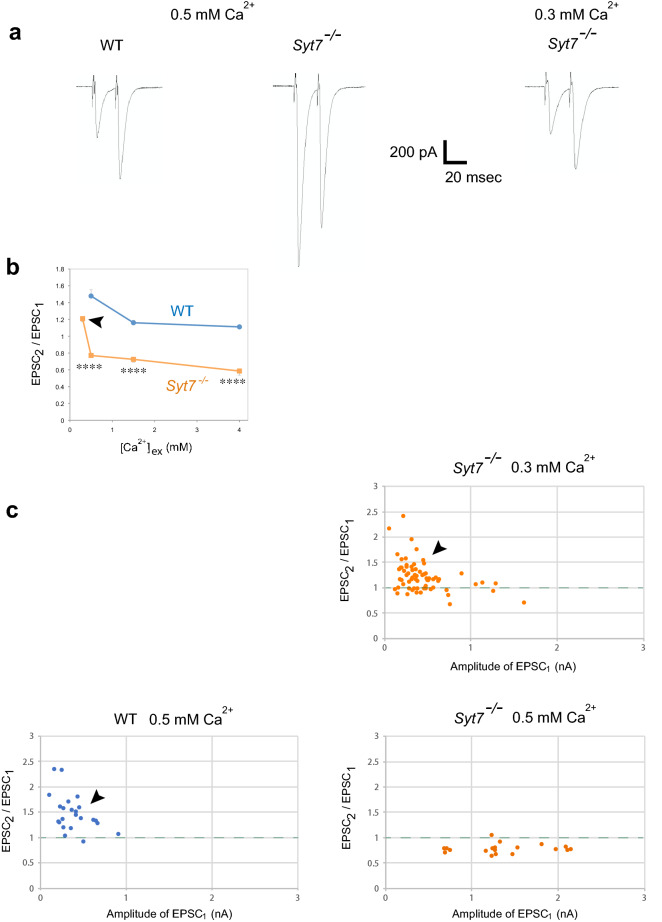
Syt7 suppresses release probability and enables paired-pulse facilitation. (**a**) Representative traces of paired-pulse facilitation with 20 ms intervals in WT and *Syt7*^*−/−*^ at 0.5 mM Ca^2+^ and in *Syt7*^*−/−*^ at 0.3 mM Ca^2+^. Twenty sets of responses to paired pulses were averaged. (**b**) Ca^2+^ dependence of facilitation ratio as 2nd pulse-induced EPSC (EPSC_2_)/1st pulse-induced EPSC (EPSC_1_) in WT and *Syt7*^*−/−*^. Note facilitation at 0.3 mM Ca^2+^ in *Syt7*^*−/−*^ (arrowhead). WT and *Syt7*^*−/−*^ were analyzed at each Ca^2+^ concentration with Mann–Whitney *U* test, and significant difference between the two groups was found at 0.5 mM Ca^2+^ (****P < 0.0001), 1.5 mM Ca^2+^ (****P < 0.0001) and 4 mM Ca^2+^ (****P < 0.0001). Ratio at 0.3 mM Ca^2+^ in WT was not quantified due to the high probability of failures in response to the 1st pulse. The numbers of samples analyzed for each genotype: (WT) 0.5 mM, 23; 1.5 mM, 10; 4 mM, 10 (*Syt7*^*−/−*^) 0.3 mM, 68; 0.5 mM, 19; 1.5 mM, 11; 4 mM, 13. (**c**) Ratios of EPSC_2_/EPSC_1_ at 0.3 mM (only *Syt7*^*−/−*^) and 0.5 mM. Responses in (**b**) were plotted with amplitude of EPSC_1_ along the X-axis. Note facilitation observed when EPSC_1_ is small (arrowheads). Error bars are SEM.

The higher release probability in *Syt7*^*−/−*^ mutants predicts decreased facilitation would occur, given synapses with stronger release probability have a lower paired pulse facilitation (PPF) ratio due to depletion of SVs during the 1st response^3,11^. Indeed, the PPF ratio in *Syt7*^*−/−*^ was much smaller than WT at 0.5 mM and 1.5 mM extracellular [Ca^2+^] (Fig. 5a,b). However, at a lower [Ca^2+^] of 0.3 mM, a positive PPF ratio was observed in *Syt7*^*−/−*^ in accordance with the smaller amplitude of the 1st EPSC (Fig. 5a,b). As shown in Fig. 5c, *Syt7*^*−/−*^ shows a relatively high PPF ratio (arrowhead in Fig. 5c) when synaptic currents are small, similar to that observed in WT. Together with the enhanced facilitation observed following Syt7 overexpression (Fig. 1d), these data indicate Syt7 levels bi-directionally gate the sign of short-term plasticity (facilitation versus depression) by controlling the levels of presynaptic output.

## Discussion

The current study indicates Syt7 is indispensable for facilitation across the physiological range of Ca^2+^ concentrations at *Drosophila* embryonic NMJs as previously shown for mammalian preparations^2^. In the absence of Syt7, the normally facilitating embryonic NMJ now displays depression. Following Syt7 overexpression, facilitation is greatly enhanced. Our data indicate the major reason for defective facilitation in *Syt7*^*−/−*^ mutants is due to loss of Syt7’s ability to suppress release, which likely causes rapid SV depletion that is non-compatible with short-term synaptic facilitation. Likewise, overexpression of Syt7 reduces SV release and allows for enhanced facilitation. This role for Syt7 contrasts with current models proposed in mammals where Syt7 is hypothesized to bind residual Ca^2+^ to directly act as a facilitation Ca^2+^ sensor. Although our data indicate Syt7 is not the primary Ca^2+^ sensor for facilitation, we cannot rule out the possibility that Syt7 has dual roles in both suppressing and facilitating SV fusion as observed for Syt1^9,21^. If Syt7 has a dual role with C2A functioning for clamping and C2B for facilitation, the null mutant would lack both properties. It is possible the lack of clamping is the dominant phenotype, with any facilitative function being masked by SV depletion at higher Ca^2+^ concentrations. Thus, we cannot rule out a Syt7-dependent component of facilitation.” However, the presence of facilitation in *Syt7*^*−/−*^ mutants at lower [Ca^2+^] (Fig. 5) indicate there is a facilitation sensor besides Syt7 that monitors residual Ca^2+^ to directly activate this form of short-term plasticity. Although we cannot completely rule out a role for residual maternally supplied Syt7 at the embryonic stage in *Syt7*^*−/−*^ mutants, there is no evidence from RNA profiling studies (Flybase) that indicate Syt7 is present at earlier stages of embryonic development prior to nervous system formation. Thus, it is unlikely residual Syt7 could sustain normal levels of facilitation as observed in low [Ca^2+^], consistent with the enhanced synaptic transmission observed across a broad [Ca^2+^] range in *Syt7*^*−/−*^ mutants. Given facilitation is also present in low [Ca^2+^] in *Syt7*^*−/−*^ mutants at the 3rd instar stage when any maternal contribution would be depleted^13^, we conclude that facilitation can occur in the complete absence of Syt7 under conditions where the initial response is reduced.

A key advantage of the *Drosophila* embryonic NMJ preparation is the ability to unambiguously monitor the absolute baseline values of synaptic strength even in high [Ca^2+^] using the non-contracting *Mhc* mutant. In this regard, it is clear that synaptic transmission at *Drosophila* embryonic NMJs is much stronger in *Syt7*^*−/−*^ mutants at all Ca^2+^ concentrations tested. Moreover, the stronger transmission is due to higher release probability rather than an increased number of releasable SVs. Thus, our data predict that higher release probability leads to a lower facilitation ratio secondary to vesicle depletion^3,11^. The precise mechanisms by which Syt7 suppresses SV release to enable facilitation will require further study. Beyond a potential clamping function for Syt7, the protein could alter local Ca^2+^ buffering or cause increased Ca^2+^ influx that could contribute to elevated SV release. Syt7 does not localize to SVs and may instead act from the plasma membrane^22^ or internal membrane compartments^13^, allowing for several potential mechanisms for Syt7 to suppress release. Ca^2+^ binding to the C2A and C2B domains of Syt1 have been shown to have distinct functions in SV release, with C2B playing a dominant role in triggering SV fusion and C2A acting to clamp release^9,23^. It is unclear if the C2A and C2B domains of Syt7 act similarly in *Drosophila* or have independent functions compared to Syt1. One possibility is that the C2A domain of Syt7 suppresses SV fusion and the C2B domain facilitates release, similar to Syt1. Structure function studies of Syt7 should help elucidate this biology in *Drosophila*, similar to our prior studies of Syt1 function.

As suppression of SV release by Syt7 is dose-dependent^13^ (Fig. 1b), increasing levels of Syt7 would elevate the ratio of facilitation as shown in Fig. 1a–d. These results suggest the degree of facilitation across distinct neuronal populations may be set by Syt7 levels similar to a potentiometer. Our analysis of *Syt7*^*−/−*^ nulls, heterozygotes and overexpression lines support such a model that changes release and short-term plasticity in a graded fashion. Depending on whether a synapse is facilitative or depressive^11^, Syt7 expression could be modulated to gate plasticity to the level that most benefits the local circuit, similar to how Syt1 and Syt2 levels variably control release synchronicity across neuronal populations^20^. Indeed, the squid giant synapse is facilitative only when Ca^2+^ is lowered from normal saline (artificial sea water)^24,25^, similar to *Syt7*^*−/−*^ mutants, suggesting synapses that normally depress may have reduced levels of Syt7. Indeed, recent evidence suggests that species-specific differences in presynaptic plasticity in rodents is linked to the levels of Syt7^26^. In shrews, the levels of Syt7 are lower in hippocampal CA3 synapses and they show reduced presynaptic plasticity. In contrast, Syt7 levels are much higher in mice, with their CA3 output synapses displaying far greater forms of presynaptic plasticity. *Drosophila* adults^27^ and 3rd instar larvae^28^ also have less facilitative NMJs than embryonic NMJs. This difference may contribute to the distinct effects of Syt7 on clamping spontaneous SV release that is observed between embryonic and 3rd instar NMJs^13^. Mammalian studies identified redundant functions for Syt1 and Syt7 in clamping spontaneous fusion at inhibitory synapses^29^. While reductions in Syt7 levels alone did not increase spontaneous SV release, removal of both Syt1 and Syt7 enhanced mini frequency to a far greater level that loss of Syt1 alone. In addition, a Syt7 transgene was able to rescue the elevated miniature frequency in *Syt1* mutants. These differences in clamping properties were attributed to an insufficient level of Syt7 expression compared to Syt1. Differences in the Syt1/Syt7 ratio between *Drosophila* 3rd instar and embryonic NMJs may also contribute to distinct effects on spontaneous SV clamping observed in *Syt1* and *Syt7* mutants at these distinct developmental stages. In conclusion, controlling expression level of Syt7 provides an attractive mechanism for activity-dependent presynaptic scaling of release probability as a homeostat for both presynaptic output and short-term facilitation, similar to postsynaptic scaling mechanisms previously described for chronic forms of synaptic plasticity^30^.

## Methods

### *Drosophila* strains

*Drosophila melanogaster* were cultured on standard medium at 25 °C. The *Syt7*^*M2*^ null mutant was used in this study^13^. *Syt7*^*M2*^ has an insertion of a Minos transposon into the second exon of the *Syt7* that generates a premature stop codon before the C2A domain, resulting in loss of Syt7 protein by Western analysis^13^. We observed similar results for *Syt7*^*M1*^, an independent null mutant generated via CRISPR-Cas9 (data not shown). All electrophysiological recordings were carried out in the background of a null mutant of muscle-specific *myosin heavy chain* (*Mhc*^*1*^) as previously described^7^. The *Mhc*^*1*^ mutant had no observed effect on synapse formation, neurotransmitter release, or postsynaptic glutamate receptor clustering^7,8^. *Syt1* null mutants were generated by crossing *Syt1*^*N13*^, an intragenic Syt1 deficiency^31^, with *Syt1*^*AD4*^, which truncates Syt1 before the transmembrane domain^19^. These null alleles were recombined with a chromosome containing *Mhc*^*1*^. Mutant second chromosomes carrying *Mhc*^*1*^ or both *Mhc*^*1*^ and *Syt1* alleles were placed over a *CyO* balancer containing *Actin*-driven GFP or *Dfd*-driven GFP (Bloomington Stock Center) to allow unambiguous identification of embryos with *Syt1* null and *Mhc*^*1*^ backgrounds. UAS transgenes were expressed using a GAL4 driver under control of the pan-neuronal *elav* promoter^32^ on the third chromosome. All lines carried the *w*-background to allow recognition of the *[w*^+^*]* marker in transgenic strains. Syt7 overexpression UAS lines have been previously described^13^.

### Electrophysiological analysis

All electrophysiology experiments were carried out as described previously^8,9^. Briefly, synaptic currents were recorded with the patch-clamp technique in whole-cell configuration from embryonic muscle fiber 6 at segments A2–A4 that were maintained at a holding potential of − 60 mV. Embryos were aged 21–24 h after fertilization and recorded in HL3.1 saline solution^33^ (in mM: NaCl, 70; KCl, 5; MgCl_2_, 5.5; NaHCO_3_, 10; trehalose, 5; sucrose, 115; Hepes–NaOH, 5; pH 7.2), using an Multiclamp 700B amplifier (Axon Instrument/Molecular Device) at 23–25 °C. External saline solutions with various concentrations of Ca^2+^ were prepared by replacing MgCl_2_ with CaCl_2_. The internal solution in patch pipettes contained: (in mM) KCl, 155; ATP, 2; EGTA, 5; HEPES–KOH, 10, pH 7.1. Before recording, embryos were dissected in Ca^2+^-free HL3.1 and treated for 1 min with 0.35 mg/mL collagenase (type IV; Sigma) in 0.1 mM Ca^2+^ saline solution. For nerve stimulation, a small part of an intact (uncut) motor nerve was sucked into a suction electrode containing bath solution at its site of emergence from the CNS, and 1.5 μA of positive current was delivered for 1 ms. For extensive electrical isolation, a negative glass electrode with a blunt end was loosely attached to the nerve at a more distal site than that of the suction electrode. Clampex 10.7 in the pCLAMP10.7 package (Molecular Devices) was used for data acquisition and data were analyzed with Clampfit 10.7 in the pCLAMP10.7 (Molecular Devices).

For hypertonic-stimulated release, 500 mM sucrose dissolved in Ca^2+^-free HL-3.1 saline solution was included in a puff pipette with a 1-μm tip. The pipette was placed in close vicinity of the boundary between muscle fibers 6 and 7 where the NMJ forms. Hypertonic solution was puffed using positive pressure for 3 s. Slow responses originating from electrically coupled muscle fibers were excluded in subsequent analysis in all genotypes. These experiments were performed in Ca^2+^-free saline to avoid enhancements of presynaptic release mediated by retrograde signaling downstream of postsynaptic Ca^2+^ influx^6,34^. Miniature analysis was performed with 1.5 mM Ca^2+^ HL3.1 containing 3 μM tetrodotoxin (TTX). Through the “Gap free” recording of Clampex, miniature release was counted for 10 min for each cell and reported as a frequency per minute.

Statistical analysis was performed using Prism6 software (GraphPad). Since the variation of data between animals or cells was small compared with the larger variation in data from one cell or one animal, results were combined and the number of events recorded are shown as N.
